# Assessing the efficacy of saline flush in frequency-domain optical coherence tomography for intracoronary imaging

**DOI:** 10.1007/s00380-023-02340-y

**Published:** 2023-12-08

**Authors:** Masahiro Kimura, Teruki Takeda, Yasushi Tsujino, Yuichi Matsumoto, Masayuki Yamaji, Tomoko Sakaguchi, Keiko Maeda, Hiroshi Mabuchi, Tomoyuki Murakami

**Affiliations:** Department of Cardiovascular Medicine, Koto Memorial Hospital, 2-1, Hiramatsu-Cho, Higashiomi-Shi, Shiga, 527-0134 Japan

**Keywords:** Contrast, Heparinized saline, Intracoronary imaging, Optical coherence tomography, Percutaneous coronary intervention

## Abstract

**Background:**

The increased amount of contrast media in frequency-domain optical coherence tomography (FD-OCT) imaging during percutaneous coronary intervention (PCI) has raised potential concerns regarding impairment of renal function.

**Objectives:**

This study aimed to evaluate the effectiveness of heparinized saline flush in FD-OCT-guided PCI and identify clinical factors contributing to optimal image quality.

**Methods:**

We retrospectively collected 100 lesions from 90 consecutive patients, and a total of 200 pullbacks were analyzed for the initial and final evaluation in which saline was used as the flushing medium.

**Results:**

The study population had a mean age of 73, with 52% having chronic kidney disease (CKD). The median amount of contrast used was 28 ml, and no complications were observed associated with saline flush OCT. Imaging quality was then categorized as excellent, good, or unacceptable. Among the total runs, 87% demonstrated clinically acceptable image quality, with 66.5% classified as excellent images and 20.5% classified as good images. Independent predictors of excellent images included lumen area stenosis ≥ 70% (adjusted odds ratio [OR] 2.37, 95% confidence interval [CI] 1.02–5.47, P = 0.044), and the use of intensive flushing (adjusted OR 2.06, 95% CI 1.11–3.86, P = 0.023) defined as a deep engagement of guiding catheter (GC) or a selective insertion of guide extension catheter (GE). Intensive flushing was performed in 60% of the total pullbacks, and it was particularly effective in improving image quality in the left coronary artery (LCA).

**Conclusion:**

The use of saline flush during FD-OCT imaging was safe and feasible, which had a benefit in renal protection with adequate imaging quality.

## Introduction

Over the decades, advancements in device technology such as drug-eluting stents, drug-coated balloons, and debulking tools have significantly improved long-term clinical results of PCI. Accurate assessment of plaque features, vessel preparation and lesion expansion, and identification of complications are essential for determining the appropriate treatment strategy and ensuring procedural success. FD-OCT has emerged as a powerful tool for intravascular visualization and assessment of coronary pathology [1]. FD-OCT utilizes near-infrared light to provide high-resolution, cross-sectional images up to 10 µm [2], and enables detailed visualization of plaque morphology, fibrous cap thickness, lipid content, calcium deposits, intraplaque microvessels, and luminal dimensions [3–5]. However, developments in OCT technology have led to the adoption of contrast agents for improved blood clearance and enhanced image quality. Despite the benefits of contrast agent-based OCT imaging, concerns have arisen regarding its potential adverse effects of contrast-induced nephropathy, particularly in patients with renal impairment [6]. Consequently, there is a growing need to explore alternative flushing mediums that can achieve efficient blood cell removal without relying on contrast agents.

In recent years, literature has emerged on the use of heparinized saline as a flush medium in FD-OCT imaging, reporting clinically applicable image quality and measurements compared to contrast agents [7–9] However, the influence of patient, lesion, and procedural characteristics on the image quality of saline flush OCT has not been sufficiently explored. Therefore, in this study, we aimed to evaluate the effectiveness of saline flush OCT with a presentation of representative cases and investigate factors contributing to optimal image quality.

## Methods

### Study population

**Fig. 1:**
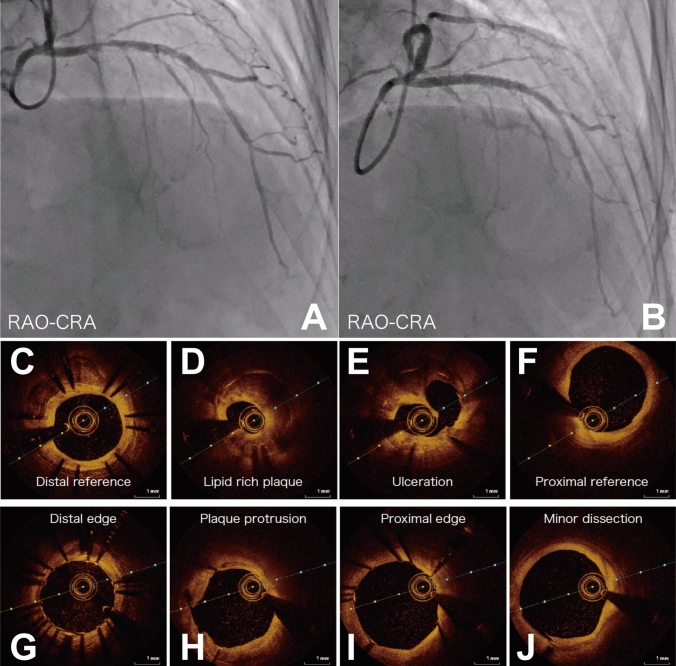
A case of PCI with saline flush OCT. Coronary angiography of pre-PCI (**A**) and post-PCI (**B**). Representative images of saline flush OCT at initial (**C-F**) and final (**G-J**) pullback. *CRA* cranial view; *OCT* optical coherence tomography; *RAO* right anterior oblique

This was a retrospective cohort study of OCT-guided PCI with saline flush at Koto Memorial Hospital between January 2020 and August 2021. In this study period, 827 patients underwent PCI for 998 lesions. Among 952 lesions in 800 patients treated with imaging devices, OCT was used for 133 lesions in 116 patients. 100 lesions in 90 patients were assessed using saline as flushing medium both in the initial and final evaluation (Fig. 1). This study was conducted in accordance with the Declaration of Helsinki, and the Institutional Ethics Committee of the Koto Memorial Hospital approved the study protocol. Consent was obtained from all participants through an opt-out methodology due to the retrospective design of the study.

### OCT procedure with flushing of heparinized saline

OCT was performed using Dragonfly Optis or Dragonfly Opstar imaging catheter (Abbott, North Chicago, IL, USA) with the preset length of 75 mm for 2.1 s. For blood clearance, 10–20 mL of heparinized 0.9% saline was injected manually into coronary arteries using 20 mL luer lock syringe. Before OCT pull back, approximately 10 mL of heparinized saline was puffed to evaluate clearance and remove the intracatheter blood. If a clear image was not obtained by this test shot, a deep engagement of guiding catheter (GC) or a selective insertion of guide extension catheter (GE) was attempted to improve image quality, which were termed as “intensive flushing” in this study. Pullbacks were conducted manually, and saline was pushed out until adequate length of images around target lesions was acquired although maximum of 375 frames were obtained for each run (0.2 mm per frame). Pullback length was described as an obtained image length during saline flush.

Imaging quality was assessed using cross-sectional images and categorized into three groups as excellent, good, and unacceptable quality. Excellent images were characterized by completely clear lumen outline for 360° of circumference, except for guidewire artifacts, in the entire pullback length. Good images were characterized by visible lumen angle ≥ 270° and the length of unclear images was less than 2 mm within the pullback, which were clinically acceptable for devising a PCI strategy. Unacceptable images were clinically unusable due to the invisible contour other than the above status.

### Data collection

Demographic, clinical, angiographic, and procedural data were collected from hospital records or electronic databases at our hospital. Lesion complexity was categorized according to the American College of Cardiology (ACC)/American Heart Association (AHA) classification.

### Statistical analysis

Categorical variables were reported as numbers and percentages, and compared using the Chi-squared test or Fisher’s exact test. Continuous variables were expressed as mean ± standard deviation, or medians and interquartile ranges, and compared using Student’s *t* test or one-way ANOVA. A logistic regression model was used to identify independent predictors for facilitating excellent images. The multivariable logistic regression analysis included variables based on the results of univariable analysis. Statistical significance was set at P < 0.05. Data were analyzed using the JMP version 16.0 software (SAS Institute, Inc., Cary, NC, USA) or R software version 3.6.2.

## Results

### Patient, lesion, and procedural characteristics

Tables 1 and 2 summarize the baseline clinical characteristics. The mean age of the entire study population was 73 years, 77% were male, and 52% had CKD. Most of the procedures were performed using 6 Fr GC through a distal radial or conventional radial approach. Although 74% of the procedures were ad-hoc PCI, the median amount of contrast used was 28 ml. Culprit vessels were left anterior descending (LAD), left circumflex (LCX), and right coronary arteries (RCA) in 41%, 20%, and 39%, respectively. There were 32% of long lesions more than 20 mm length. No complication was observed during saline flush.

**Table 1:** Clinical characteristics of the patients

Characteristic	Value
Total patients	90
Age, years	73.1 ± 7.6
Male	69 (77)
Body mass index, kg/m^2^	24.4 ± 3.0
Comorbidity/risk factor	
Hypertension	77 (86)
Chronic kidney disease	47 (52)
Hemodialysis	2 (2)
Diabetes	18 (20)
Previous PCI history	68 (76)
LV dysfunction (EF < 40%)	6 (7)
Atrial fibrillation	10 (11)
Laboratory findings	
Serum creatinine	0.94 [0.77–1.17]
eGFR, mL/min/1.73m^2^	58.8 [46.7–69.2]
Hemoglobin, g/dL	13.7 [12.4–14.7]
BNP, pg/mL	30.0 [14.4–51.4]
Approach site	
Distal RA	61 (68)
RA	24 (27)
FA	5 (6)
Guiding catheter size	
6 Fr	85 (94)
5 Fr	5 (6)
Ad-hoc PCI	67 (74)
Procedure time, min	71 [55–87]
Contrast, mL	28 [22–39]
Exposure dose, mGy	1066 [712–1526]

Data are presented as the mean ± SD, median [interquartile range], or number (%)

*BNP* B-type natriuretic peptide; *EF* ejection fraction; *eGFR* estimated glomerular filtration rate; *FA* femoral artery; *LV* left ventricular; *PCI* percutaneous coronary intervention; *RA* radial artery

**Table 2:** Clinical characteristics of the lesions

Characteristic	Value
Total lesions	100
Lesion location	
LAD	41 (41)
LCX	20 (20)
RCA	39 (39)
Type B2/C lesion	63 (63)
Lesion length > 20 mm	32 (32)
Tortuous lesion	2 (2)
Calcified lesion	34 (34)
True bifurcation lesion	12 (12)
In-stent restenosis	25 (25)
Devices	33 (2)
DES	80 (80)
DCB	20 (20)

Data are presented as number (%)

*DCB* drug-coated balloon; *DES* drug-eluting balloon; *LAD* left anterior descending artery; *LCX* left circumflex coronary artery; *RCA* right coronary artery

### Case presentation

A 72-year-old male with a history of multiple PCIs and CKD (eGFR 47 mL/min/1.73 m^2^) underwent coronary angiography due to recurrent chest pain. He received an everolimus-eluting stent implantation for stent-edge restenosis of a prior sirolimus-eluting stent at the proximal segment of LAD. The representative saline flush OCT images with a selective insertion of GE are described in Fig. 1. At the initial evaluation, the detailed information of plaque features was achieved with saline flush. After stent implantation, final procedural success was ensured using saline flush OCT. Saline flush OCT produced adequate images of pathological structure like lipid-rich plaque, ulceration, plaque protrusion, and dissection.

### Assessment of imaging quality

A comparison of procedure-related characteristics and factors affecting coronary blood flow between initial and final pullbacks is shown in Table 3. Systolic and diastolic blood pressure was higher in initial pullbacks than those in final pullbacks, whereas heart rate was similar between these two groups. There was no significant difference of saline volume, pullback length, and the use of intensive flushing between initial and final pullbacks. The median volume of saline was 15 ml in both initial and final pullbacks. Approximately 60% of saline flush was performed with intensive flushing, one-third of which was a deep engagement of GC and the rest was the use of GE. When intensive flushing was performed, the amount of injected saline was significantly lower than without intensive flushing (12 [12–14] ml and 20 [18–20] ml, median [interquartile range], respectively, P < 0.0001).

**Table 3:** Procedural characteristics and imaging quality between initial and final pullbacks

Characteristic	Initial pullback (*n* = 100)	Final pullback (*n* = 100)	*P* value
Saline volume, mL	15 [12–20]	15 [12–20]	1.0
Pullback length, mm	54 [43–65]	49 [41–60]	0.20
Systolic blood pressure, mmHg	138 [122–152]	112 [102–131]	< 0.0001
Diastolic blood pressure, mmHg	68 [60–77]	62 [57–70]	0.006
Mean blood pressure, mmHg	91 [82–101]	80 [72–88]	< 0.0001
Heart rate, /min	70 [63–78]	70 [64–79]	0.77
Intensive flushing	59 (59)	61 (61)	0.77
GC deep engagement	22 (22)	24 (24)	0.74
GE use	37 (37)	37 (37)	1.0
Imaging quality			
Excellent	70 (70)	63 (63)	0.29
Good	21 (21)	20 (20)	0.86
Unacceptable	9 (9)	17 (17)	0.09

Data are presented as the median [interquartile range], or number (%)

*GC* guiding catheter; *GE* guide extension catheter

**Fig. 2:**
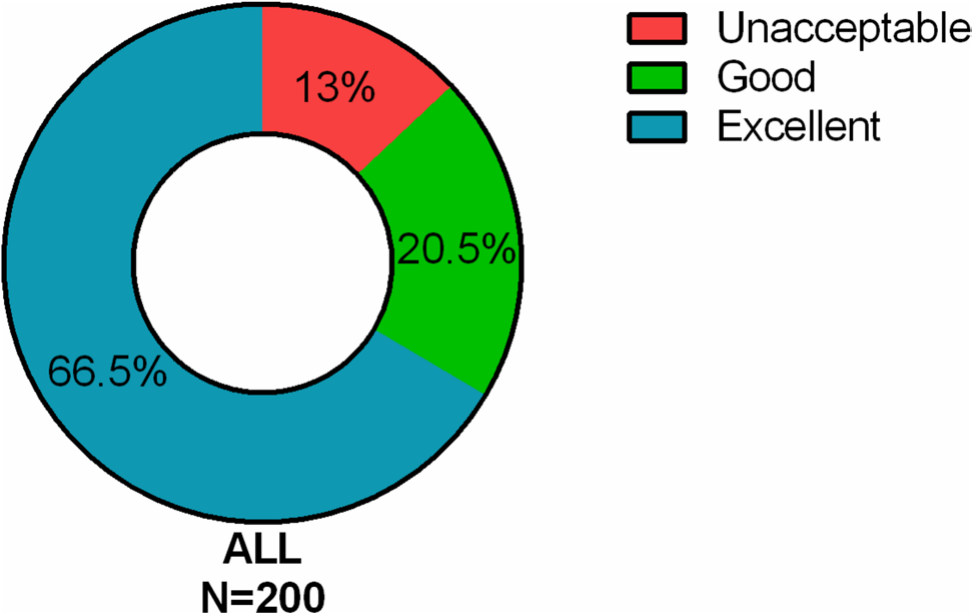
Distribution of imaging quality in total pullbacks

**Fig. 3:**
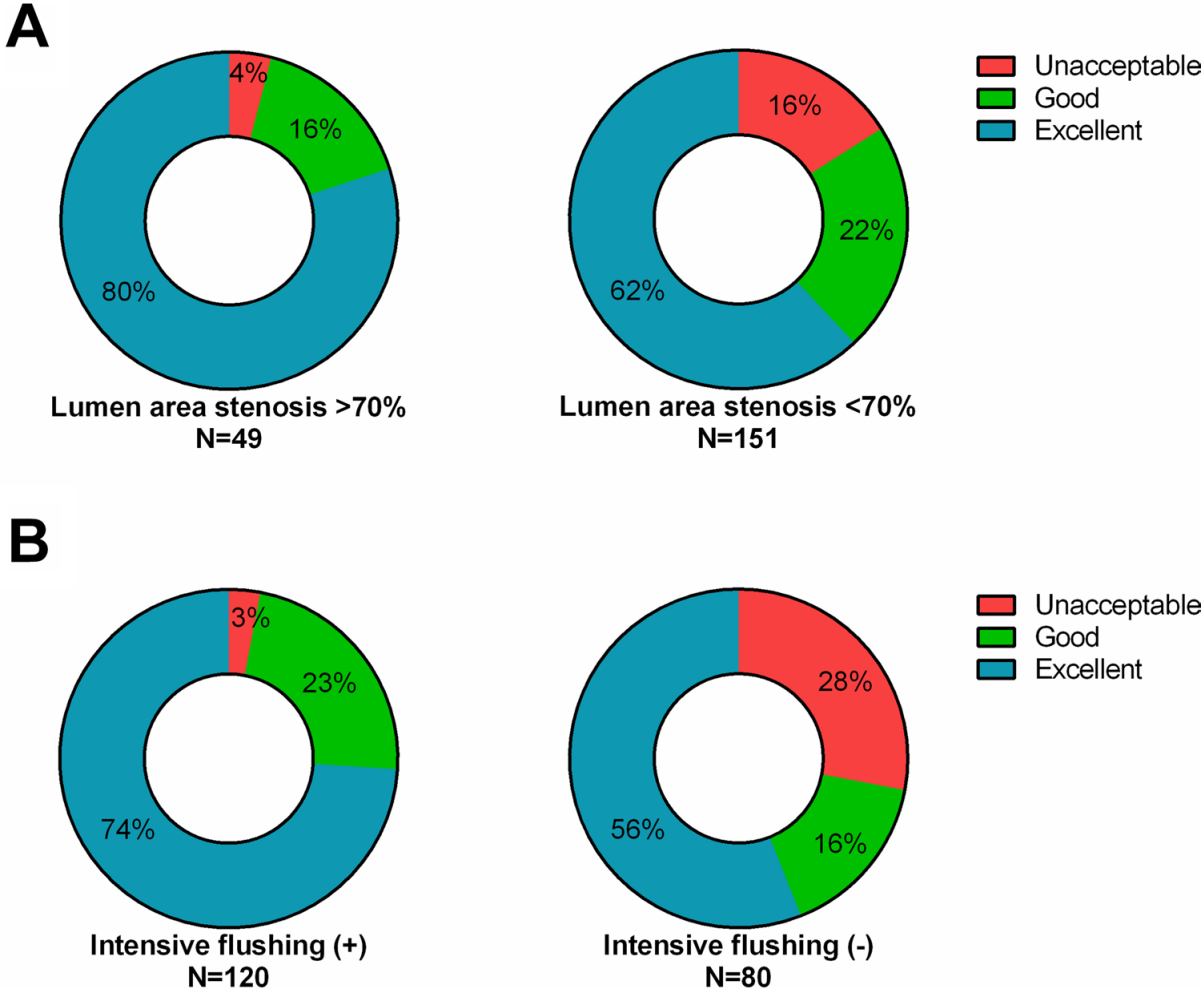
Differences of imaging quality based on **A** lumen area stenosis and **B** intensive flushing

**Fig. 4:**
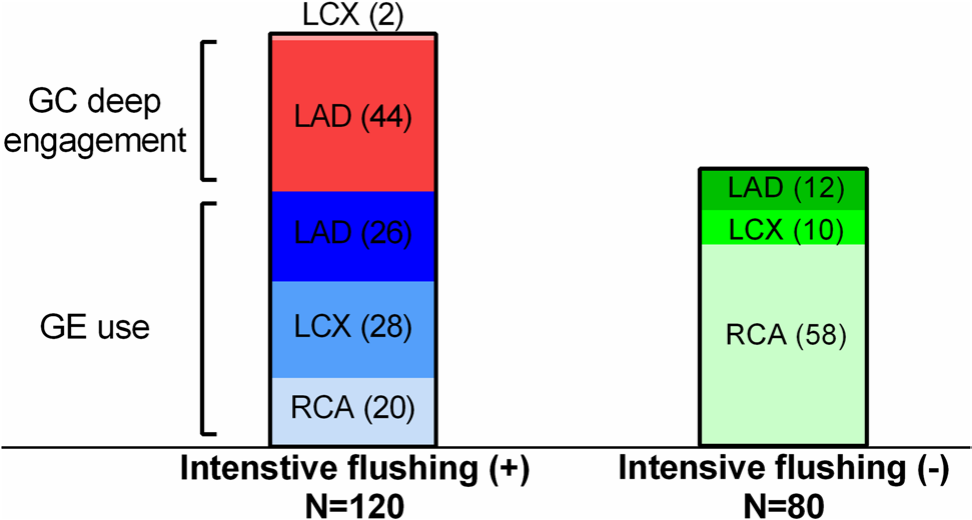
The procedural details of intensive flushing. *LAD* left anterior descending artery; *LCX* left circumflex coronary artery; *RCA* right coronary artery

The result of imaging quality is described in Fig. 2. Clinically acceptable images including excellent and good quality were obtained in 91% of initial pullbacks and 83% of final pullbacks. No significant difference in imaging quality was found between initial and final pullbacks (Table 3). The predictors of acquiring excellent images were evaluated using univariable and multivariable analysis (Table 4). Lumen area stenosis over 70% and the use of intensive flushing were independent predictors of excellent images. Excellent images were obtained in 80% of lesions with lumen area stenosis ≥ 70%, and in 74% of lesions assessed with intensive flushing (Fig. 3). The detail of 120 pullbacks with intensive flushing is described in Fig. 4. Most of cases with deep engagement of GC were performed to assess LAD lesions, while the use of GE was almost evenly applied to all three coronary vessels. The amount of injected saline was larger in RCA than LAD and LCX due to differences in the prevalence of intensive flushing. On the other hand, the distribution of attained images was comparable among three coronary vessels (Table 5).

**Table 4:** Univariable and multivariable analysis of predictors for excellent images in saline flush OCT

Variables	Unadjusted OR (95% CI)	*P* value	Adjusted OR (95% CI)	*P* value
Age	0.97 (0.93–1.01)	0.15		
Male	0.81 (0.40–1.61)	0.57		
Mean blood pressure	1.005 (0.99–1.03)	0.61		
Heart rate	0.994 (0.97–1.02)	0.63		
Anemia (Hb < 11.0 mg/dL)	0.53 (0.24–1.19)	0.12		
Type B2/C lesion	1.64 (0.90–2.99)	0.11		
Lumen area stenosis ≥ 70%	2.36 (1.10–5.10)	0.028	2.37 (1.02–5.47)	0.044
Intensive flushing	2.14 (1.18–3.89	0.013	2.06 (1.11–3.86)	0.023

*OCT* optical coherence tomography

**Table 5:** Comparison of procedural characteristics and imaging quality among the pullbacks of each coronary artery

Characteristic	LAD (*n* = 82)	LCX (*n* = 40)	RCA (*n* = 78)	*P* value
Intensive flushing	70 (85)	30 (75)	20 (26)	< 0.0001
Saline volume, mL	14 [12–15]	12 [10–19]	19 [15–20]	< 0.0001
Without intensive flushing	20 [16–20]	20 [20]	20 [18–20]	0.18
With intensive flushing	12 [12–15]	11 [8–12]	14 [12–15]	0.0002
Imaging quality				0.25
Excellent	49 (60)	29 (73)	55 (71)	
Good	20 (24)	9 (23)	12 (15)	
Unacceptable	13 (16)	2 (4)	11 (14)	

Data are presented as the median [interquartile range], or number (%)

*LAD* left anterior descending artery; *LCX* left circumflex coronary artery; *RCA* right coronary artery

## Discussion

The main findings of this study are summarized as follows: 1) OCT with saline flush were safe and effective to minimize contrast medium; 2) saline OCT offered adequate images for clinical assessment; 3) deep engagement of GC or use of GE were beneficial for improving images of saline OCT.

### Clinical application of saline flush OCT

The study population in this study had a mean age of 73, and approximately half of the patients had CKD. This aligns with the findings from the previous Japanese multicenter registry including patients with a mean age of 69.4 and a prevalence of 40% for CKD, which also reported an increasing trend in the average age of patients undergoing PCI and the proportion of patients with renal dysfunction [10]. FD-OCT is an immensely valuable and rapidly emerging tool in guiding the treatment strategy of PCI. However, it has been reported that the use of contrast agents for flushing in FD-OCT procedures leads to an additional total volume of 33 ml of contrast agent being required [11], which raises concerns about contrast-induced nephropathy and its adverse impact on clinical outcomes. Therefore, minimizing the use of contrast agents is necessary, particularly in patients with renal impairment using FD-OCT in PCI procedures.

An alternative flushing medium, low-molecular-weight dextran (LMWD), has been widely used in clinical settings. Several studies have investigated the feasibility and imaging quality of LMWD-based OCT imaging, demonstrating comparable results to contrast agent-based imaging [12, 13]. However, it is important to note that despite the overall safety profile, rare cases of anaphylactoid reactions like Kounis syndrome [14, 15] or acute kidney injury [16] have been reported with dextran use. Considering that heparinized saline is a more cost-effective and commonly used medium in PCI procedures, it is reasonable to explore its use as a flush solution in FD-OCT imaging. Previous reports on saline flush OCT in carotid [17] and lower limb [18] arteries have suggested superior blood clearance efficiency compared to coronary arteries, and saline flush OCT in coronary arteries has been somewhat challenging.

Recently, several case reports highlighting the effective clinical application of saline flush with the reduction of the total amount of contrast have been documented [19, 20], and prospective observational studies have reported findings on saline flush OCT [7–9]. These studies primarily focus on comparing the images obtained from contrast flush and saline flush. Nalin and Ankush performed saline flush OCT by manual injection in 27 patients, and reported that good-quality images were obtained in 61% of cases, with 88.1% of images being clinically usable [7]. They also reported that the measured parameters were comparable to those obtained with contrast flush in a cohort of 10 patients [8]. Similarly, Ankita et al. achieved good-quality images in approximately 80% of cases from 20 patients using saline flush with an automatic delivery system, with approximately 95% of images being clinically usable. They further demonstrated a strong correlation (R^2^ = 0.92) between dimensional measurements obtained from saline and contrast [9]. A common observation from their reports is that RCA tends to yield better images compared to LCA, and no complications were observed with saline flush OCT. In this study, we analyzed a larger sample size (90 patients) and observed no complications with saline flush OCT. We obtained sufficient images for determining PCI strategy in 91% of initial pullbacks and 83% of final pullbacks. Although the presence of the bifurcation in the LCA might reduce blood cell clearance efficiency with saline flush, our study suggests that image quality can be improved by appropriately employing deep engagement of the GC or using GE for selective flushing. In fact, we implemented intensive flushing, incorporating these techniques, in 60% of the entire pullback, with 83% of these cases being performed in the LCA. While the application rate of intensive flushing varied between the LCA and RCA, the consistent imaging quality suggests that saline flush OCT is feasible for any of the three coronary arteries.

### Safety and feasibility of saline flush OCT

In general, higher viscosity fluids such as contrast medium can effectively displace blood and minimize blood scattering [21, 22]. However, blood cell clearance efficiency is influenced by various factors such as blood flow rate, fluid velocity, injection pressure, vascular anatomy and size, and injection site. Heparinized saline exhibits lower viscosity compared to contrast agents or LMWD [13], allowing for increased injection flow rates and potentially improved blood cell removal efficiency, especially in the cases of manual injection. In the present study, physiological factors that could potentially influence coronary artery flow rate, such as blood pressure, heart rate, and anemia, did not significantly affect image quality. However, consistent with the previous report [9], there were differences in imaging quality between the diastolic and systolic phases. It is of note that image quality could be improved by adjusting the timing and attempting another flush in some cases, taking into account the differences in the timing of the cardiac cycle. On the other hand, high-pressure and high-flow injections have the potential to increase intra coronary artery pressure, thereby raising the risk of ventricular tachyarrhythmias and coronary artery dissection. In this study, as in previous studies [7, 8], transient electrocardiographic changes were observed following saline flush. Although most cases of them exhibited T-wave inversion and QT prolongation, there was no instances of ventricular arrhythmias. However, attention should be paid to the possibility of ventricular arrhythmias, especially in cases with frequent premature ventricular contractions. Furthermore, there were no observed complications including coronary artery dissection in any cases. The amount and pressure of the flushing solution can vary depending on the particular coronary artery under examination, the area of interest within the vessel, vessel size, and the patient’s heart rate. Consequently, manual injection may be preferred over an automated injector, as it allows for delicate control over the required volume and push force to obtain high-quality images. The fundamental approach for achieving this is to maintain close monitor of the OCT pullback while manually administering heparinized saline.

Several challenges have been identified in the use of OCT for PCI. One is its application in coronary ostial lesions. According to previous studies, OCT can be a reasonable option for the treatment of LMT lesions, but this is the case for mid or distal LMT lesions [23–25]. The use of OCT for lesions at the ostial region of the LMT remains challenging. A recent report has described the use of a Telescope GE (Medtronic Cardiovascular, Santa Rosa, CA, USA) that allows partial transmission of near-infrared light to perform OCT imaging of ostial lesions [26]. Another limitation involves the usage of OCT in highly stenotic lesions where wedging the OCT catheter makes blood clearance difficult, often requiring the PUSH procedure [27]. Whether these techniques can be applied in conjunction with saline flush OCT warrants further investigation.

### Study limitations

There are several limitations to consider in this study. First, it was conducted as a retrospective design. The decision to utilize OCT as the imaging modality was left to the discretion of the operators, which could introduce selection bias and limit the generalizability of the findings. Second, the study was conducted at a single center, which may limit the external validity of the results. Third, there was the lack of a comparative group using alternative imaging modalities or flushing medium, which had a potential concern regarding the clinical suitability of images that were deemed as “good” by our judgment but had some areas of blurriness. Fourth, all PCI procedures performed using saline flush OCT in this study achieved acute success; however, this study did not evaluate long-term clinical outcomes. Future prospective studies with larger sample sizes and long-term follow-up are needed to address these limitations.

## Conclusion

In conclusion, the current study collectively supported the use of saline flush in clinical practice and its efficacy in minimizing contrast agent usage. Factors contributing to obtaining satisfactory images were also investigated, and a deep engagement of GC or a selective insertion of GE using was beneficial in achieving better images for saline flush FD-OCT.

## Data Availability

The availability of data and materials for this study is ‘Available on request’.
